# Function and Global Regulation of Type III Secretion System and Flagella in Entomopathogenic Nematode Symbiotic Bacteria

**DOI:** 10.3390/ijms25147579

**Published:** 2024-07-10

**Authors:** Xiyin Huang, Chen Li, Ke Zhang, Kunyan Li, Jiajie Xie, Yuyuan Peng, Meifang Quan, Yunjun Sun, Yibo Hu, Liqiu Xia, Shengbiao Hu

**Affiliations:** State Key Laboratory of Developmental Biology of Freshwater Fish, Hunan Provincial Key Laboratory of Microbial Molecular Biology, College of Life Science, Hunan Normal University, Changsha 410081, China; xiyinhuang@hunnu.edu.cn (X.H.); 202270142917@hunnu.edu.cn (K.L.); xialq@hunnu.edu.cn (L.X.)

**Keywords:** entomopathogenic nematode symbiotic bacteria, T3SS, flagella, T6SS

## Abstract

Currently, it is widely accepted that the type III secretion system (T3SS) serves as the transport platform for bacterial virulence factors, while flagella act as propulsion motors. However, there remains a noticeable dearth of comparative studies elucidating the functional disparities between these two mechanisms. Entomopathogenic nematode symbiotic bacteria (ENS), including *Xenorhabdus* and *Photorhabdus*, are Gram-negative bacteria transported into insect hosts by *Steinernema* or *Heterorhabdus*. Flagella are conserved in ENS, but the T3SS is only encoded in *Photorhabdus*. There are few reports on the function of flagella and the T3SS in ENS, and it is not known what role they play in the infection of ENS. Here, we clarified the function of the T3SS and flagella in ENS infection based on flagellar inactivation in *X. stockiae* (*flhDC* deletion), T3SS inactivation in *P. luminescens* (*sctV* deletion), and the heterologous synthesis of the T3SS of *P. luminescens* in *X. stockiae*. Consistent with the previous results, the swarming movement of the ENS and the formation of biofilms are dominated by the flagella. Both the T3SS and flagella facilitate ENS invasion and colonization within host cells, with minimal impact on secondary metabolite formation and secretion. Unexpectedly, a proteomic analysis reveals a negative feedback loop between the flagella/T3SS assembly and the type VI secretion system (T6SS). RT-PCR testing demonstrates the T3SS’s inhibition of flagellar assembly, while flagellin expression promotes T3SS assembly. Furthermore, T3SS expression stimulates ribosome-associated protein expression.

## 1. Introduction

The T3SS serves as a micro-syringe and is prevalent in Gram-negative bacteria, facilitating the direct delivery of effector proteins into the cytoplasm of host cells. This process is enabled by the formation of a transport conduit spanning the inner and outer membranes of both the bacterium and the host cell [1,2]. Structurally, the T3SS comprises key components such as substrates, chaperones, translocators, and effectors [3,4]. Effectors play a pivotal role in modulating the host’s global response, thereby influencing the outcome of infection [5,6]. In contrast, flagella represent a ubiquitous class of biological appendages found across bacterial species. Comprising three main components, the basal body, flagellar hook, and flagellar filament, flagella serve as a versatile motility apparatus. The basal body, traversing both inner and outer bacterial membranes, acts as an anchor and power source [7]. Meanwhile, the flexible flagellar hook facilitates the transmission of power between the basal body and the flagellar filament [8]. Due to their structural resemblance, it is widely hypothesized that flagella and the T3SS share evolutionary origins [3].

While the T3SS and flagella exhibit structural similarities, their functional roles diverge significantly. Current research on these systems predominantly focuses on model organisms like *Escherichia coli* and *Salmonella* [9]. In these organisms, flagella predominantly operate in a peritrichous manner, capable of rotating both counterclockwise (CCW) and clockwise (CW). This rotational switching modulates bacterial perceptions of external environmental cues and signal molecules, thereby facilitating bacterial movement and biofilm formation [10]. Conversely, the T3SS specializes in precisely targeting host cells and deploying effector proteins to disrupt the host’s immune defenses. As a result, the T3SS primarily facilitates the process of infection and colonization within the host [11].

The expression and assembly of flagella and the T3SS in bacteria are tightly regulated processes crucial for infection and colonization [12]. During bacterial infection, these processes can be divided into three distinct phases, each with its own specific requirements for flagella and T3SS expression. In the initial stage of infection, directional movement is essential for the bacterium to reach its target site. Flagella play a vital role in providing the necessary motility, and thus, their expression is continuously up-regulated during this phase [13,14]. However, once the bacterium reaches its destination, the directional mobility facilitated by the flagella becomes unnecessary. Moreover, flagellin, a major component of flagella, can trigger immune responses in the host through recognition receptors such as TLR-5, NRLC4, NAIP5/6, potentially leading to the failure of the infection [15]. Consequently, to evade host immune detection and facilitate virulence, the bacterium down-regulates flagellin expression and initiates the assembly of the T3SS upon contact with the host. The T3SS serves as a specialized virulence release system that is rapidly activated upon interacting with the host [4]. Consequently, during this phase, the pathogen actively suppresses flagellin expression and prioritizes the assembly and activation of the T3SS. Once activated, the T3SS secretes effectors into the host, which interfere with signal transduction and inflammatory pathways, thereby promoting successful infection and colonization [16]. Consequently, T3SS expression remains high throughout the infection process to sustain interference with host cellular processes. It is worth noting that both flagella and T3SS assembly are energetically costly processes for the bacterium [17,18]. However, this regulatory mechanism not only promotes successful infection but also conserves energy resources by strategically allocating them to the most crucial virulence factors. Furthermore, functional differences between the T3SS and flagella-mediated mechanisms are significant. While flagella primarily facilitate bacterial motility and initial contact with the host, the T3SS is specifically designed for the delivery of virulence factors directly into host cells, enabling the manipulation of host cellular processes to the bacterium’s advantage [19].

The genomes of *Xenorhabdus* and *Photorhabdus* harbor numerous unidentified non-ribosomal peptide synthetases and polyketide synthases, which are delivered into the host, holding significant potential for biocontrol applications [1]. Thus, comprehending the roles of the T3SS and flagella in ENS not only aids in elucidating ENS infection mechanisms but also fills critical research gaps concerning the T3SS and flagella in this context.

## 2. Results

### 2.1. Flagella Play a Pivotal Role in Governing Both Swarming Motility and Biofilm Formation in Xenorhabdus

**Figure 1:**
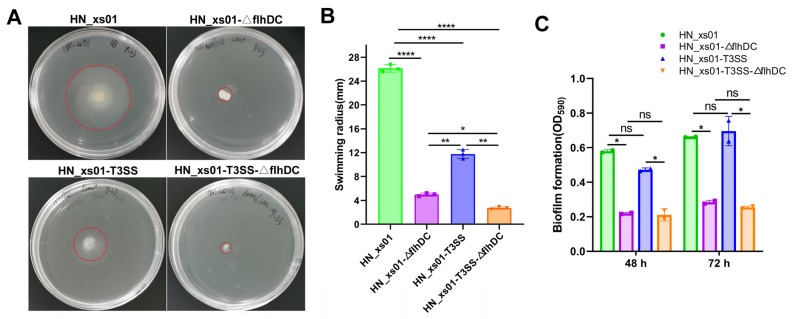
Effects of flagella and T3SS on motility and biofilm formation of *X. stockiae*. (**A**) The results of swarming motility of different *X. stockiae* strains after growing them on 0.4% LB agar plates for 24 h. (**B**) Statistical results of swimming radius. The experiment was repeated three times for each group, and the error bars are derived from the standard deviation of the mean values of the three independent experiments. (**C**) Biofilm formation of different *X. stockiae* strains. Biofilm was measured according to the description in Materials and Methods. The error bars represent the standard deviation from the average of the two independent experiments, and each experiment was set up with 5 replicate holes for each strain. * *p* < 0.05; ** *p* < 0.01; **** *p* < 0.0001; ns, not significant.

Our previous investigations have demonstrated the functional capability of the T3SS from *P. luminescens* TT01 following its expression in *X. stockiae* HN_xs01. FlhDC, identified as the master operon of the flagellar system in Xenorhabdus, orchestrates the expression of flagellin [20]. Thus, we opted to disrupt *flhDC* to generate a flagellar-deficient strain of HN_xs01. A flocking motion analysis revealed a significant reduction in the swimming radius of HN_xs01 subsequent to *flhDC* deletion. Intriguingly, the expression of the T3SS not only failed to enhance the swarming motility of HN_xs01 but also attenuated its movement capacity (Figure 1A,B). Additionally, we evaluated the biofilm-forming potential of these strains. The findings indicate that only the flagella-deficient strains, namely HN_xs01-ΔflhDC and HN_xs01-T3SS-ΔflhDC, exhibited minimal biofilm formation at both 48 h and 72 h (Figure 1C). These outcomes collectively suggest that the coordinated movement and biofilm development of HN_xs01 are solely mediated by flagella, with the T3SS playing no discernible role in these processes.

### 2.2. T3SS and Flagella Collaboratively Facilitate the Invasion and Colonization of ENS While Leaving Secondary Metabolites Unaffected

**Figure 2:**
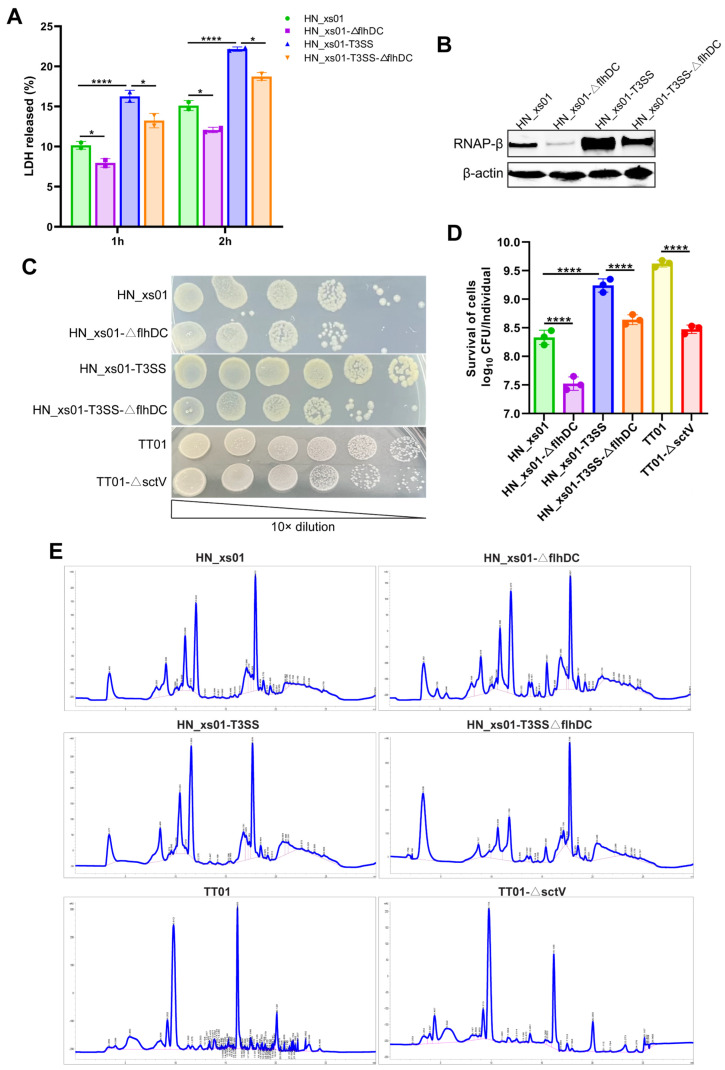
Effects of flagella and T3SS on the infection ability and stimulating metabolites of ENS. (**A**) Detection of extracellular LDH content after co-culture of different *X. stockiae* strains with HeLa cells. The error bars represent the standard deviation from the average of the two independent experiments, and each strain is set up with 5 replicate wells in each experiment. (**B**) Invasion efficiency of different *X. stockiae* strains after co-culture with HeLa cells for 8 h. (**C**) Colonization of different strains of ENS in vivo after 24 h of injection of *H. armigera*. (**D**) Statistics on colonization ability of different ENS strains. The error bars represent the standard deviation from the average of the 3 independent experiments, and there were 5 replicates for each strain in each experiment. (**E**) The synthesis of secondary metabolites in different ENS strains after 72 h culture. * *p* < 0.05; **** *p* < 0.0001.

The T3SS and flagella are often associated with the infection and colonization of pathogens. Adhesion or firm attachment to host cells marks the initial phase of infection [21]. Hence, we assessed the adhesion impact of ENS and HeLa cells by measuring extracellular lactate dehydrogenase (LDH) levels following the co-cultivation of HN_xs01 with HeLa cells. Our findings revealed a significant reduction in extracellular LDH release from the HeLa cells upon flagellar inactivation, whereas the expression of the T3SS led to a noteworthy increase in extracellular LDH release (Figure 2A). This underscores the involvement of both flagella and the T3SS in the adhesion of ENS to HeLa cells. Subsequently, we delved deeper into the invasion capability of HN_xs01 into HeLa cells. Following 8 h of co-cultivation of HN_xs01 with HeLa cells, we quantified the invasion using Western blotting of RNAP-β. The results exhibited a considerable decrease in detected RNAP-β upon flagellar deletion, whereas the T3SS expression significantly augmented the detected RNAP-β levels (Figure 2B). This implies that both the T3SS and flagella play pivotal roles in promoting the invasion of HN_xs01 into HeLa cells.

The ultimate aim of pathogen infection is to establish colonization within the host. We simulated the unique infection pattern of ENS by injecting them into the blood cavity of *Helicoverpa armigera*. SctV serves as a pivotal gating protein within the T3SS’s machinery, regulating substrate selection and contributing to energy conversion, crucial for the assembly of a functional T3SS [22,23]. Consequently, we employed SctV knockout in *P. luminescens* TT01 to generate a strain lacking a functional T3SS. All ENS were conferred gentamicin resistance before being injected into cotton bollworms. Following a 24 h injection period, the dissected worms underwent screening on gentamicin resistance plates. Remarkably, the number of clones formed by HN_xs01 lacking flagella and TT01 lacking a functional T3SS was notably diminished on the resistance plate, while the abundance of clones generated by HN_xs01 expressing T3SSs significantly increased (Figure 2C,D). This underscores the indispensable role of the T3SS and flagella in the in vivo colonization of ENS.

ENS exhibit a capacity for abundant metabolite production, and given the pivotal roles of flagella and the T3SS as critical channels for material exchange, they may influence the secondary metabolite profile of ENS. Consequently, we extracted and concentrated the 72 h fermentation broth of all of the ENS, followed by a high-performance liquid chromatography (HPLC) analysis. Interestingly, no significant alterations were observed in the final metabolites upon flagellar deletion, T3SS heterologous expression, or T3SS deletion in the ENS (Figure 2E).

### 2.3. Both T3SS and Flagella Play Crucial Roles in Mediating the Chemotaxis of ENS

**Figure 3:**
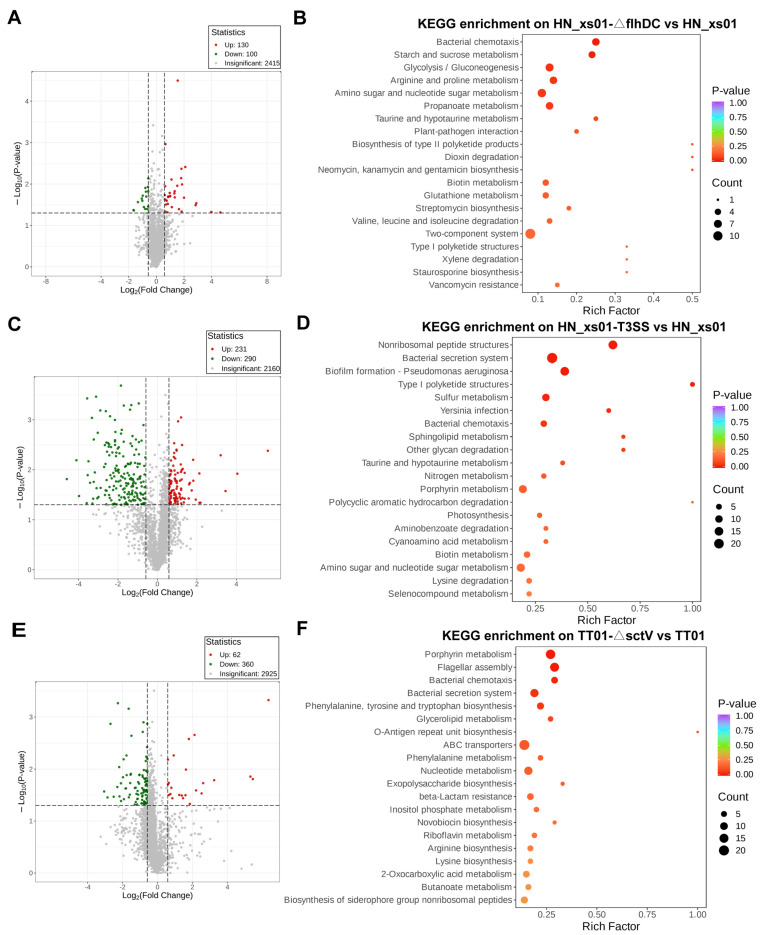
Proteomic analysis between strains with altered flagella or T3SS functional activity and wild-type strain. (**A**) Volcano map of differentially expressed proteins between flagellar inactivated HN_xs01-flhDC and wild-type strain. (**B**) Bubble map of differentially expressed proteins in the first 20 KEGG enrichment pathways of HN_xs01-ΔflhDC and HN_xs01. (**C**) Volcano map of differentially expressed proteins between HN_xs01-T3SS and wild-type strain. (**D**) Differentially expressed protein bubble maps of the first 20 KEGG enrichment pathways of HN_xs01-T3SS and HN_xs01. (**E**) Volcano plot of differentially expressed proteins between T3SS-inactivated TT01-ΔsctV and wild-type strain. (**F**) Differentially expressed protein bubble maps of TT01-ΔsctV and TT01’s top 20 KEGG enrichment pathways. In the volcanic map, the red point represents up-regulation, the green point represents down-regulation, and the gray point represents no significant change. The enrichment factor in the bubble diagram refers to the ratio of the number of differentially expressed proteins in the pathway to the number of all annotated proteins in the pathway.

To delve deeper into their functional mechanisms, we conducted an intracellular proteomics analysis of ENS. The proteins identified in the engineered strain were compared with those of the wild-type strain to identify differential proteins. Subsequently, these proteins with significant differences underwent KEGG enrichment to assess the function of flagella and the T3SS in ENS. When the flagella of HN_xs01 were inactivated, a total of 230 differentially expressed proteins emerged, among which 130 were significantly up-regulated and 100 were significantly down-regulated (Figure 3A). These proteins primarily participate in ENS chemotaxis, as well as in the metabolism and synthesis of various substances (Figure 3B). Upon the heterologous expression of the T3SS in HN_xs01, 521 proteins exhibited significant expression differences, with 231 being up-regulated and 290 being down-regulated (Figure 3C). These differential proteins are mainly involved in ENS chemotaxis, the metabolism and degradation of various substances, and the pathogen’s infectivity (Figure 3D). Furthermore, when the functional T3SS in TT01 was deactivated, 422 proteins exhibited significant expression differences, with only 62 up-regulated and 360 down-regulated (Figure 3E). These proteins are primarily associated with bacterial chemotaxis, flagellar assembly, substance metabolism and synthesis, and protein transport (Figure 3F). These findings underscore the importance of both flagella and the T3SS in mediating ENS chemotaxis, with their malfunction impacting the synthesis and metabolism of various substances. Moreover, the greater number of differentially expressed proteins resulting from changes in T3SS activity suggests that T3SS-mediated biological functions are more intricate and diverse.

### 2.4. T3SS and Flagella Inhibited the Expression of T6SS

**Figure 4:**
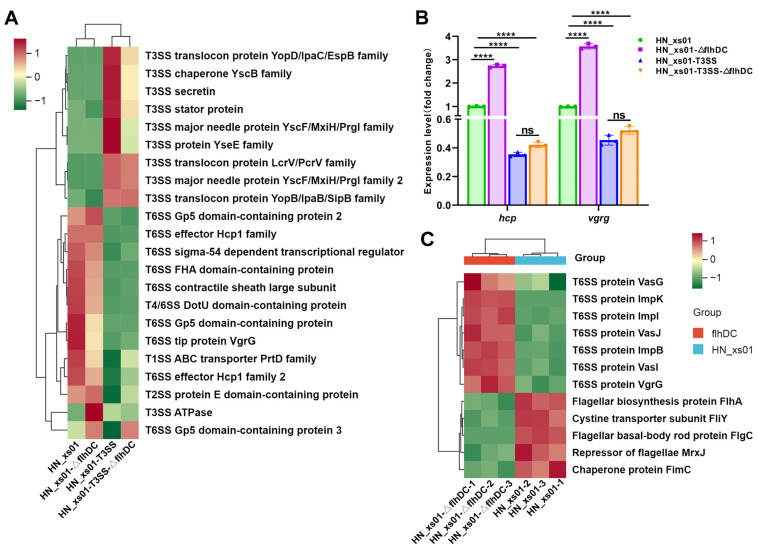
Effects of flagella and the T3SS on T6SS expression in *X. stockiae*. (**A**) Cluster heat map of secretory system-related proteins in intracellular proteomics of strains. (**B**) The expression of Hcp and VgrG was detected by RT-PCR testing. The error bars represent the standard deviation of the average value of the 3 independent experiments, and each experiment was set up with 3 replicate holes for each strain. (**C**) Cluster heat map of T6SS and flagella-related proteins in HN_xs01-ΔflhDC and HN_xs01 extracellular proteomes. The red box represents up-regulated expression, and the green box represents down-regulated expression. **** *p* < 0.0001; ns, not significant.

It has previously been documented that flagella, the T3SS, and other secretion systems share interrelations and mutually regulate each other [24,25]. To investigate whether this regulatory dynamic extends to ENS, we conducted a comprehensive screening and clustering of proteins associated with the secretion system within ENS. Our clustering analysis revealed that the T3SS was notably expressed in HN_xs01, and its expression significantly down-regulated the expression of proteins related to T6SS (Figure 4A). To further corroborate this intriguing observation, we conducted an RT-PCR analysis. VgrG trimer formation is pivotal for T6SS assembly, and Hcp serves dual roles as both a structural component of T6SS and an effector contributing to virulence [26,27,28]. Hence, we selected *vgrg* and *hcp* as indicators to assess T6SS expression. Consistent with our proteomic findings, when the T3SS was expressed, the expression levels of VgrG and Hcp exhibited a significant decrease. Additionally, upon the inactivation of flagella in HN_xs01, we observed a marked increase in the expression of *vgrg* and *hcp* (Figure 4B). This led us to hypothesize that flagella assembly also hampers T6SS expression. Subsequently, we conducted an extracellular proteomic analysis on wild-type and flagella-inactivated engineered strains of HN_xs01. Through meticulous screening and clustering, we observed a substantial rise in the secretion of T6SS-related proteins in the extracellular environment following flagellar inactivation, while flagella-associated proteins showed a notable decrease (Figure 4C). Collectively, these findings suggest that the presence of the T3SS and flagella concurrently suppresses T6SS expression.

### 2.5. Flagella Promote the Assembly of the T3SS, While the T3SS Negatively Regulates the Expression of Flagellin

**Figure 5:**
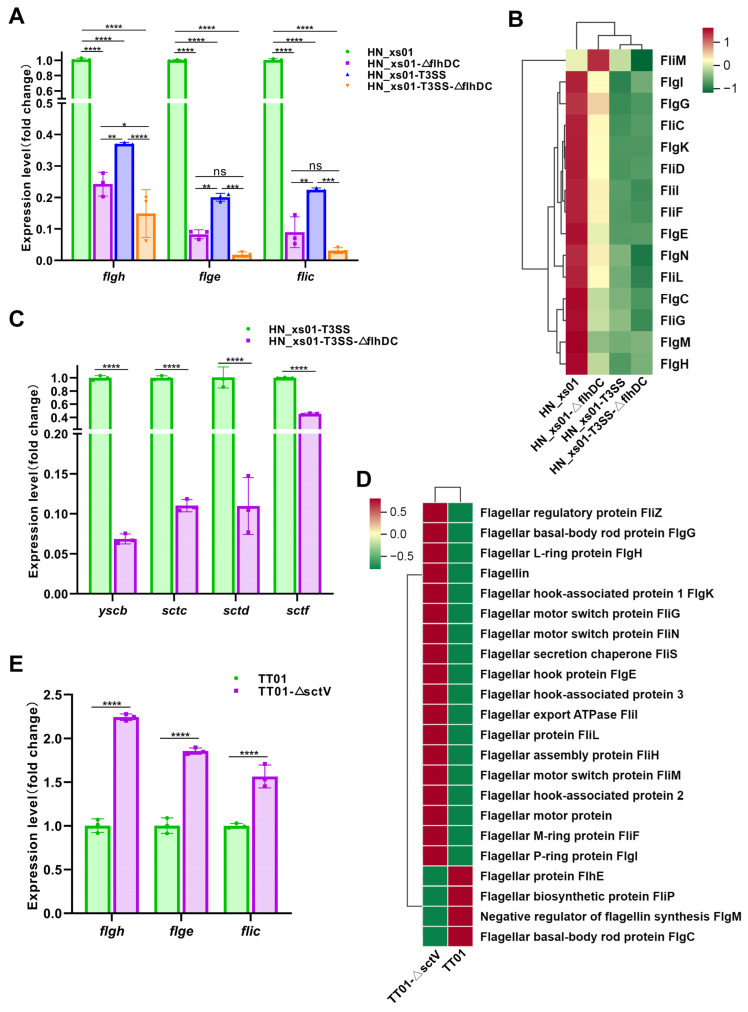
Interaction between flagella and T3SS in ENS. (**A**) The expression of flagella-related proteins in *X. stockiae* was detected by RT-PCR testing. (**B**) Cluster heat map of flagella-related protein expression in *X. stockiae* intracellular proteome. (**C**) The expression of T3SS-related proteins in *X. stockiae* was detected by RT-PCR testing. (**D**) Cluster heat map of flagella-related protein expression in *P. luminescens* intracellular proteome. (**E**) The expression of flagella-related proteins in *P. luminescens* was detected by RT-PCR testing. The error bars in (**A**,**C**,**E**) represented the standard deviation of the mean values of the 3 independent experiments, and there were 3 replicate wells per strain. The red box in (**B**,**D**) indicates up-regulated expression, and the green box indicates down-regulated expression. * *p* < 0.05, ** *p* < 0.01; *** *p* < 0.001; **** *p* < 0.0001; ns, not significant.

Upon observing that the expression of T3SS-related proteins was diminished in flagella-inactivated HN_xs01 (Figure 4A), we hypothesized that the presence of flagella might promote the expression of the T3SS. To investigate this hypothesis, we utilized YscB (chaperone protein), SctC (outer membrane ring subunit), SctD (inner membrane ring subunit), and SctF (needle-like structural subunit) of the T3SS as evaluation criteria [29], comparing their expression differences between HN_xs01-T3SS and HN_xs01-T3SS-ΔflhDC. The results from the RT-PCR analysis indicated a significant decrease in the expression levels of *yscb*, *sctc*, *sctd*, and *sctf* in flagella-inactivated strains (Figure 5C). This finding aligns with the results of the proteomic analysis, suggesting that flagella indeed promote the expression and assembly of the T3SS.

Subsequently, we conducted a screening and clustering analysis of flagellin-related proteins in the proteomics dataset, revealing that the expression of the T3SS significantly down-regulated the expression of flagellin in HN_xs01 (Figure 5B). Similarly, we validated the expression of FlgH (L-ring matrix protein), FlgE (flagellar hook protein), and FliC (flagellar filament protein) through RT-PCR testing [30]. Consistently, the results mirrored those of the proteomics analysis, demonstrating a down-regulation of *flgh*, *flge*, and *flic* expressions to varying degrees upon T3SS activation (Figure 5A). Furthermore, we conducted a parallel analysis in TT01, yielding results in line with our expectations. Specifically, upon the inactivation of the T3SS in TT01, there was a significant up-regulation of the expression of flagella-related proteins (Figure 5D,E). These findings collectively indicate that within ENS, flagella play a crucial role in promoting the assembly of the T3SS, while the T3SS exerts a negative feedback regulatory effect on flagella.

### 2.6. T3SS Enhances the Expression of Ribosome-Associated Proteins

**Figure 6:**
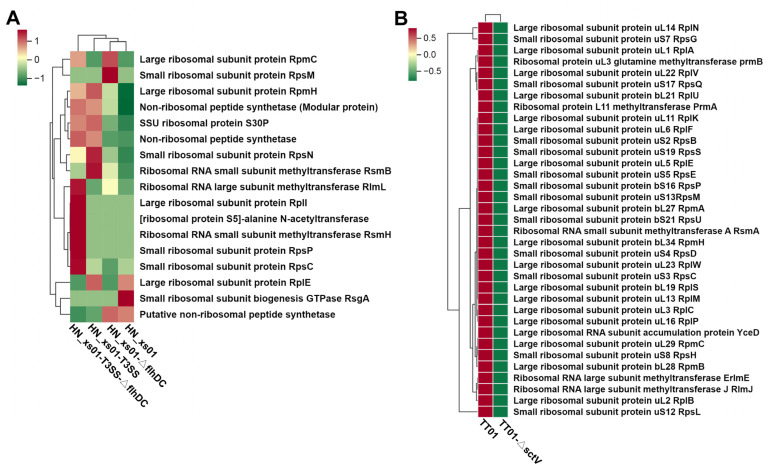
Effects of the T3SS on ribosome-associated proteins in ENS. (**A**) Cluster heat map of ribosome-associated protein expression in the intracellular proteome of *X. stockiae*. (**B**) Cluster heat map of ribosome-related protein expression in *P. luminescens* intracellular proteome. The red box indicates up-regulated expression, and the green box indicates down-regulated expression.

Both the flagellar system and the T3SS are intricate machineries, often involving the synthesis and transportation of numerous proteins. In prokaryotic cells, protein production commonly relies on the ribosomal machinery [3]. Therefore, we conducted a further analysis on ribosome-associated proteins within the ENS. In HN_xs01, the absence of a flagellar system did not appear to influence the expression of ribosomal proteins. However, upon T3SS activation, there was a significant up-regulation observed in the majority of ribosomal subunit proteins (Figure 6A). A parallel analysis conducted in TT01 yielded consistent results; upon the inactivation of the T3SS, there was a notable decrease in the expression levels of ribosomal subunit proteins (Figure 6B). These findings suggest a positive correlation between T3SS activation and the expression of ribosomal proteins within the ENS, independent of the flagellar system’s influence.

## 3. Discussion

**Figure 7:**
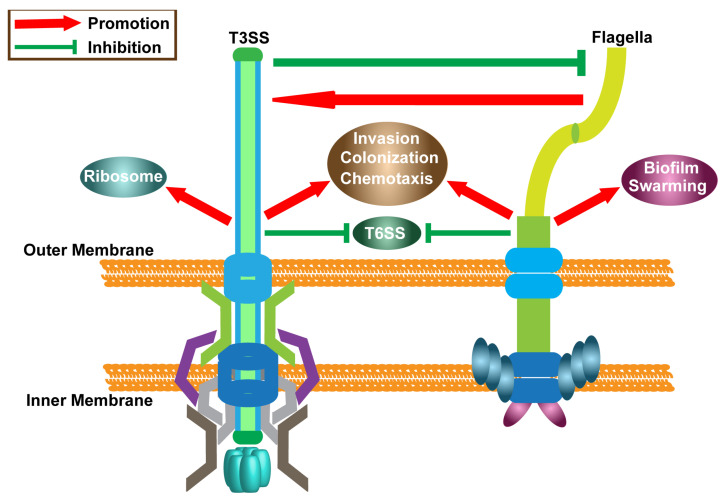
The function and global regulation model of T3SS and flagella in ENS. The red arrow indicates a promotional effect, and the green line segment indicates an inhibitory effect.

Microbial infection is a multifaceted process wherein pathogens must overcome numerous barriers imposed by the environment and the host. Consequently, bacterial evolution has engendered a diverse array of mechanisms aimed at breaching these barriers to facilitate infection [31,32]. In this study, employing gene knockout techniques and the heterologous expression of the T3SS, we conducted a comparative analysis to delineate the respective contributions of flagella and the T3SS to ENS infection and colonization. Our findings confirm the essential role of flagella in the formation of biofilms and the swarming motility of ENS. Moreover, both flagella and the T3SS were deemed indispensable for the invasion and infection of ENS. Through an integrated approach incorporating bacterial intracellular and extracellular proteomics analyses, as well as RT-PCR validation, we elucidated the intricate regulatory network governing flagella, the T3SS, and the T6SS in the context of ENS infection. An erichment analysis of differentially expressed proteins corroborated the regulatory influence of flagella and the T3SS on ENS chemotaxis. Furthermore, through the screening and clustering of directional proteins, coupled with RT-PCR validation, we established the inhibitory effect of the T3SS and flagella on the T6SS. Notably, our study unveils a complex feedback loop between flagella and the T3SS: flagella assembly promotes T3SS expression, while the T3SS further promotes the expression of ribosomal-related proteins and forms a negative feedback regulation of flagella (Figure 7).

*Xenorhabdus* and *Photorhabdus* must synchronize with the life cycle of Steinernema or *Heterorhabdus*, entering the nematode body at precisely the right moment and maintaining symbiosis with the nematode. This distinctive symbiotic model places exceptionally high demands on bacterial movement and infection capabilities [33,34]. Notably, both *Xenorhabdus* and *Photorhabdus* feature peripheral flagella, possibly as an adaptation to their unique survival requirements [35,36]. The formation of bacterial biofilms often hinges on the motility of flagella [37]. Our findings corroborate this phenomenon within the context of ENS infections, in which flagella inactivation significantly diminishes *Xenorhabdus*’s ability to form biofilms. While flagellar-driven motility in ENS enhances their competitive adaptability during infection, it is not an absolute prerequisite for the release of virulence factors [38]. Consequently, the observed decrease in *Xenorhabdus*’s infection capacity following flagellar inactivation in our study may be attributed to a decline in competitive adaptability.

The T6SS, akin to the T3SS, functions as a protein nano-syringe equipped with transmembrane channels [39]. However, unlike the T3SS, the T6SS boasts a wider host spectrum, effectively targeting both prokaryotic and eukaryotic cells, thereby facilitating intraspecific competition and host infection [40]. Consequently, the physiological processes governed by the T6SS are notably diverse, encompassing survival, transmission, colonization, evasion, and manipulation of the host’s innate immune response [41,42,43]. Unlike the T3SS, which necessitates an unfolded structure for effector secretion, the T6SS can directly transport effectors extracellularly through the sheath formed by TssB/C. Nevertheless, akin to the T3SS, the assembly and operation of T6SS constitute resource-intensive endeavors within bacterial systems [44,45]. Hence, a stringent regulatory framework is essential to ensure the orderly execution of bacterial physiological activities. A class of transcriptional regulators, including RpoN and RpoS, play pivotal roles in sensing changes in the growth environment and modulating gene expression to adapt to these fluctuations [46,47,48]. RpoN is extensively involved in critical physiological processes such as cofactor biosynthesis, nucleotide metabolism, oxidative phosphorylation, amino acid biosynthesis and metabolism, flagellar assembly, bacterial chemotaxis, and secretion systems. Consequently, the absence of RpoN results in a cascade of defective phenotypes, including slowed growth and the inability to form flagella [49]. Similarly, RpoS serves as the principal regulator of the stress response, playing indispensable roles in flagellar formation, virulence, and quorum sensing [50,51]. Additionally, our findings indicate that alterations in flagellar or T3SS functionality impact bacterial chemotaxis, as well as the synthesis and metabolism of nucleotides and various amino acids. These effects may also be attributed to the intervention of transcriptional regulators.

The intricate global regulation orchestrated by transcriptional regulators of flagellar assembly and bacterial secretion systems has garnered increasing attention [52]. In *Plesiomonas shigelloides*, the deletion of RpoN led to the suppression of flagellar system expression, as well as genes related to T2SS and T6SS, while leaving the expression of T3SS genes unaffected [53]. Conversely, in *Yersinia pseudotuberculosis*, RpoS not only promotes flagella formation but also directly stimulates the expression of T6SS4, bolstering bacterial resistance [54]. Our research underscores the presence of a complex interplay among flagella, the T3SS, and the T6SS in ENS, yet the precise regulatory mechanisms demand further exploration. It is plausible that this intricate global regulation involves potential transcriptional regulators. Considering the unique lifestyle of ENS, they serve not only as symbionts that share mutual benefits with nematodes but also as pathogens that suppress insect growth. The living conditions for ENS vary significantly across different stages. During the symbiotic phase, ENS experience minimal survival pressure, potentially leading to a dormant state in all virulence mechanisms. However, when ENS initiate infection alongside nematodes, they face heightened competitive pressures. During this phase, ENS may elevate the expression of theT6SS to secure a favorable niche for their survival. Hence, this coordinated global regulation across multiple systems could be pivotal for ENS in adapting to varying environmental pressures and transforming roles effectively. In summary, our investigation discerned and elucidated disparities between the flagellar system and the T3SS-mediated functions in ENS, unveiling for the first time the intricate global regulation intertwining flagella, the T3SS, and the T6SS in ENS. This meticulous and systematic regulation facilitates the infection process with enhanced efficiency and energy conservation, potentially constituting a widespread phenomenon across bacterial species.

## 4. Materials and Methods

### 4.1. Bacterial Strains, Cell Lines, and Their Growth Conditions

In this study, *X. stockiae* HN_xs01 and *P. luminescens* TT01 were used as wild-type strains and grown at 30 °C. *E. coli* DH5α was used for molecular cloning and recombinant plasmid construction, and *E. coli* MFDpir was used as the donor bacteria providing the plasmids in conjugation, both of which were grown at 37 °C. Other strains used in this study are shown in Table 1. The above strains were cultured in Luria-Bertani (LB) medium. The antibiotics used were erythromycin (100 μg/mL), chloramphenicol (30 μg/mL), gentamicin (50 μg/mL), and kanamycin (30 μg/mL). The concentrations of antibiotics were halved when used in liquids. HeLa cells were cultured in DMEM medium, supplemented with 10% heat-inactivated FBS, 100 μg/mL penicillin, and 100 μg/mL streptomycin at 37 °C.

### 4.2. Motility Assay

The overnight culture was diluted at 1:20 and grown to OD_600_ of 0.6, then diluted at 1:100 and inoculated on LB plates with 0.4% agar. The plates were incubated at 30 °C for 24 h, and then the colony radius was measured.

### 4.3. Biofilm Assays

Biofilm formation was determined according to the previously described crystal violet staining method [55]. HN_xs01 was grown overnight in liquid LB, diluted with fresh LB medium to OD_600_ of 0.2, and then transferred to a sterile 96-well plate. After static incubation at 30 °C for 48 h and 72 h, the medium was removed, and the well plate was washed twice with phosphate buffered saline (PBS). The pores were then filled with 300 μL crystal violet solution (0.1% *w*/*v*), and the plates were incubated at room temperature for 2 min. After absorbing crystal violet solution, the orifice plate was washed with PBS 3–5 times and dried at room temperature for 20 min. A volume of 200 μL ethanol solution (70% *v*/*v*) was added into the pores, and the absorbance at 590 nm was measured.

### 4.4. LDH Assays

HeLa cells were seeded in 96-well plates at a density of 5 × 10^3^ cells/well and cultured overnight at 37 °C. The overnight-cultured ENS were washed 3 times with PBS and re-suspended in serum-free cell culture medium at a standardized concentration of 1 × 10^6^ CFU/mL. The content of LDH was determined using the LDH cytotoxicity assay kit (C0016, Beyotime, Haimen, China) after co-culturing different ENS with HeLa cells for a specific duration.

### 4.5. Immunoblotting

HeLa cells were washed with ice-cold PBS and then lysed on ice with RIPA buffer (WB-0072, Beijing Dingguo Changsheng, Beijing, China) containing a protease inhibitor for 10 min to prepare a cell lysate. The protein concentration was determined using the BCA method, and an equal amount of protein sample was added to 5 × loading buffer containing β-mercaptoethanol. After mixing it, the mixture was heated at 98 °C for 10 min to fully denature the proteins. Equal amounts of proteins were loaded onto 4–10% SDS gels and transferred to PVDF membranes. To block the membranes, a solution of TBS containing 0.1% Tween-20 (TBST) with 5% skim milk was used at room temperature for 1 h and then left overnight at 4 °C or at room temperature for 2 h with a primary antibody probe. After washing the membranes with TBST, the cells were incubated with Hrp-labeled secondary antibody at room temperature for 1–2 h. The membrane was then washed with TBST and incubated with ECL Clarity Max substrate (K-12045-D50, Advansta, San Jose, CA, USA), followed by imaging with the Tanon-5200 imaging system.

### 4.6. Detection of ENS Colonization Ability

*H. armigera* were purchased from Keyun Biotechnology Co., Ltd. (Guangzhou, China) and were used for ENS injection after being grown to the fourth instar in a 28 °C incubator. During the injection procedure, 10 μL of bacterial solution was injected into the *H. armigera*’s third pair of ventral feet. To determine the colonization ability of ENS in vivo, the biomass of an overnight culture was adjusted to a standardized concentration of 1 × 10^8^ CFU/mL. After 24 h of injection, the *H. armigera* were grounded and crushed in PBS. The resulting suspension was then diluted using a gradient of 10^−1^, and 5 μL from each dilution were taken. These diluted droplets were cultured on LB agar plates containing gentamicin for 48 h, after which the bacterial colonies were counted.

### 4.7. HPLC Assays

The ENS fermentation supernatant cultured at 30 °C for 72 h was extracted with ethyl acetate and freeze-dried with SPD121P (Thermo Fisher, Waltham, MA, USA) equipment. Before performing HPLC using Agilent 1260 system (Santa Clara, CA, USA), the sample was dissolved in 1ml methanol and filtered through a 0.22 μm membrane. The samples were injected on a reversed-phase C18 column (YMC-Pack ODS-AQ, 5 μm, 4.6 × 150 mm, Shimadzu, Kyoto, Japan), equipped with Agilent 1260 HPLC system, with a cross-gradient elution of acetonitrile and methanol and UV monitoring at 210 nm.

### 4.8. Proteomics Assays

All proteomics were performed by Metware Biotechnology Co., Ltd. (Wuhan, China) For ENS intracellular proteins, the bacteria cultured for 18 h were collected, and total proteins were extracted in lysis buffer (8 M urea, containing 1 mM PMSF and 2 mM EDTA). For extracellular secreted proteins, the supernatant was collected after 24 h of culture, and 1 mM PMSF was added to concentrate the sample multiple times with a 10 kd ultrafiltration tube. The cleavage, labeling, separation, detection, and database retrieval of all proteins were completed by Metware Biotechnology Co., Ltd. (Woburn, MA, USA) All proteomic analyses are performed on the Metware Cloud platform (https://cloud.metware.cn).

### 4.9. Quantitative Real-Time PCR (RT-PCR) Assays

The bacteria were collected and treated with 0.5 mg/mL lysozyme for 5 min. The total RNA of ENS was obtained using RNA rapid extraction kit (M5105, NCM Biotech, Seattle, WA, USA). Primer ScriptTM RT Reagent Kit (RR047A, Takara, Kusatsu, Shiga, Japan) was used to perform DNA enzyme treatment and cDNA synthesis according to the instructions. ArtiCanATM SYBR qPCR Mix (TSE501, Beijing Qingke, Beijing, China) was used for RT-PCR amplification. The transcription level of the samples was determined using a 7500 Real Time PCR system (Applied Biosystems, Waltham, MA, USA). The expression level of the measured genes was calculated using 2^−ΔΔCt^ method. The primer pairs used for RT-PCR are listed in Table 2.

### 4.10. Statistical Analysis

GraphPad Prism version 8.0.1 was used for plotting and statistical analysis. All data are in line with normal distribution. Significance was assessed by one-way ANOVA, and t-test was performed using independent samples. All error bars denote mean values ± SD or SEM, as indicated in each figure legend (* *p* < 0.05; ** *p* < 0.01; *** *p* < 0.001; **** *p* < 0.0001; ns, not significant).

## Figures and Tables

**Table 1:** Bacterial strains and plasmids.

Strain or Plasmid	Genotype or Description	Source
**Strains**		
HN_xs01	wild-type *X. stockiae* HN_xs01	laboratory preservation
HN_xs01-ΔflhDC	flagella inactivation (Cm^r^)	this study
HN_xs01-T3SS	heterogeneous synthesis of the T3SS (Erm^r^)	this study
HN_xs01-T3SS-ΔflhDC	T3SS activity without flagella (Cm^r^ Erm^r^)	this study
TT01	wild-type *P. luminescens* TT01	laboratory preservation
TT01–ΔsctV	T3SS inactivation (Cm^r^)	this study
**Plasmids**		
pEVS107-T3SS	T3SS gene cluster (Erm^r^)	laboratory preservation
pRE112::flhDC	knockout flhDC (Cm^r^)	this study
pRE112::sctV	knockoutsctV (Cm^r^)	this study

**Table 2:** Primers used in this study.

Primer	Sequence	Information
X-16s-F	CAGATGGGATTAGCTAGTAG	the *16s* of *X. stockiae* HN_xs01
X-16s-R	TGCAATATTCCCCACTGCTG	the *16s* of *X. stockiae* HN_xs01
X-Flge-F	GATGGTTCAACCACTACGAC	the *flge* of *X. stockiae* HN_xs01
X-Flge-R	AGAGTACGGTCTGAGTCCAT	the *flge* of *X. stockiae* HN_xs01
X-Flgh-F	GTACTCGCATTGTCGGTACT	the *flgh* of *X. stockiae* HN_xs01
X-Flgh-R	AACAGAACCATTCGGCGCTG	the *flgh* of *X. stockiae* HN_xs01
X-Flic-F	CAACCTGTACAGCCATTGAG	the *flic* of *X. stockiae* HN_xs01
X-Flic-R	TGGCAGCAACTCTAAGAGTG	the *flic* of *X. stockiae* HN_xs01
X-Vgrg-F	CATCAATCACCACTTTCGCG	the *vgrg* of *X. stockiae* HN_xs01
X-Vgrg-R	CGATAATGAATACCGGGTAC	the *vgrg* of *X. stockiae* HN_xs01
X-Hcp-F	CTCTGCTGGATGTTCAACAC	the *hcp* of *X. stockiae* HN_xs01
X-Hcp-R	GTATTGGGTGGTGACTTAC	the *hcp* of *X. stockiae* HN_xs01
YscB-F	CAGTCGTTTAGGCCAGAAGC	the *yscb* of *X. stockiae* HN_xs01
YscB-R	CTTAGCGGGCTGGATAACAC	the *yscb* of *X. stockiae* HN_xs01
SctC-F	CTGCTTATAGCCAAGATCTG	the *sctc* of *X. stockiae* HN_xs01
SctC-R	CTTGTCGCTGACAACCACTG	the *sctc* of *X. stockiae* HN_xs01
SctD-F	GCACCTGATCTTAGAAGTCG	the *sctd* of *X. stockiae* HN_xs01
SctD-R	CACAATAACGGGCCAACTTC	the *sctd* of *X. stockiae* HN_xs01
SctF-F	TCCGCAACAACAGTGAATAC	the *sctf* of *X. stockiae* HN_xs01
SctF-R	GCTAGCAAAGCTGGGTTATC	the *sctf* of *X. stockiae* HN_xs01
P-16s-F	GAAGAAGCACCGGCTAACTC	the *16s* of *P. luminescens* TT01
P-16s-R	GATGCCATTCCCAGGTTGAG	the *16s* of *P. luminescens* TT01
P-Flge-F	CAACGACGACTAACCGAACA	the *flge* of *P. luminescens* TT01
P-Flge-R	TGCGGTTCTCATCCATCTTG	the *flge* of *P. luminescens* TT01
P-Flgh-F	CACCTTCTGTGGCAACTGCA	the *flgh* of *P. luminescens* TT01
P-Flgh-R	CCTGCAATGTAATCGTCAGG	the *flgh* of *P. luminescens* TT01
P-Flic-F	GAGCGTCTATCTTCTGGTCT	the *flic* of *P. luminescens* TT01
P-Flic-R	CTGTGCAATAGAGATACCGT	the *flic* of *P. luminescens* TT01

## Data Availability

The raw data supporting the conclusions of this article will be made available by the authors upon request.
